# Down-regulation of USP13 mediates phenotype transformation of fibroblasts in idiopathic pulmonary fibrosis

**DOI:** 10.1186/s12931-015-0286-3

**Published:** 2015-10-09

**Authors:** Jing Geng, Xiaoxi Huang, Ying Li, Xuefeng Xu, Shuhong Li, Dingyuan Jiang, Jiurong Liang, Dianhua Jiang, Chen Wang, Huaping Dai

**Affiliations:** Department of Respiratory and Critical Care Medicine, Beijing Key Laboratory of Respiratory and Pulmonary Circulation Disorders, Beijing Chao-Yang Hospital-Beijing Institute of Respiratory Medicine, Capital Medical University, Beijing, 100020 P.R. China; Department of Medical Research, Beijing Chao-Yang Hospital, Capital Medical University, Beijing, 100020 P.R. China; National Clinical Research Centre for Respiratory Medicine, Beijing Hospital, Beijing, 100730 P.R. China; Department of Medicine Pulmonary Division and Women’s Guild Lung Institute, Cedars-Sinai Medical Center, Los Angeles, CA 90048 USA; China–Japan Friendship Hospital, Beijing, 100029 P.R. China

**Keywords:** USP13, Fibroblasts, Phenotype transformation, PTEN, Idiopathic pulmonary fibrosis

## Abstract

**Background:**

Idiopathic pulmonary fibrosis (IPF) is a fatal disease characterized by fibroblastic foci and progressive scarring of the pulmonary parenchyma. IPF fibroblasts display increased proliferation and enhanced migration and invasion, analogous to cancer cells. This transformation-like phenotype of fibroblasts plays an important role in the development of pulmonary fibrosis, but the mechanism for this is not well understood.

**Methods:**

In this study, we compared gene expression profiles in fibrotic lung tissues from IPF patients and normal lung tissues from patients with primary spontaneous pneumothorax using a cDNA microarray to examine the mechanisms involved in the pathogenesis of IPF. In a cDNA microarray, we found that USP13 was decreased in lung tissues from patients with IPF, which was further confirmed by results from immunohistochemistry and western blot assays. Then, we used RNA interference in MRC-5 cells to inhibit USP13 and evaluated its effects by western blot, real-time RT-PCR, bromodeoxyuridine incorporation, and transwell assays. We also used co-immunoprecipitation and immunofluorescence staining to identify the correlation between USP13 and PTEN in IPF.

**Results:**

USP13 expression levels were markedly reduced in fibroblastic foci and primary IPF fibroblast lines. The depletion of USP13 resulted in the transformation of fibroblasts into an aggressive phenotype with enhanced proliferative, migratory, and invasive capacities. Additionally, USP13 interacted with PTEN and mediated PTEN ubiquitination and degradation in lung fibroblasts.

**Conclusions:**

Down-regulation of USP13 mediates PTEN protein loss and fibroblast phenotypic change, and thereby plays a crucial role in IPF pathogenesis.

**Electronic supplementary material:**

The online version of this article (doi:10.1186/s12931-015-0286-3) contains supplementary material, which is available to authorized users.

## Introduction

Idiopathic pulmonary fibrosis (IPF) is a progressive lethal interstitial lung disease of unknown etiology with limited effective therapies [1]. It occurs more commonly in elderly adults, and the median survival time is only 2–3 years from diagnosis, similar to many cancers [1, 2]. The pathogenic mechanisms of IPF remain unknown. However, it has been generally accepted that IPF involves aberrant wound healing in response to repetitive alveolar epithelial micro-injuries, ultimately leading to the formation of scar tissue and a severe distortion of pulmonary architecture [3]. Fibroblastic foci, the hallmark lesions of IPF, are adjacent to sites of recent alveolar injury and indicate current ongoing fibrogenesis [3, 4]. Their numbers on surgical lung biopsies are correlated with disease progression in IPF patients [5, 6].

Fibroblastic foci are characterized by the exaggerated accumulation of myofibroblasts and excessive deposition of fibrillar collagens within the alveolar wall [7]. Myofibroblasts in fibroblastic foci, which express α-smooth muscle actin (α-SMA) and have contractile properties, are responsible for excessive collagen deposition and appear to be resistant to apoptosis [8, 9]. Fibroblasts are the major contributors to the myofibroblast population in fibroblastic foci [10]. Compared with cells from normal donors, fibroblasts from IPF patients show more aggressive biologic behaviors, including enhanced proliferation and augmented migration and invasion [11–13], reminiscent of cancer cells. Emerging evidence indicates that the abnormal behaviors of fibroblasts in IPF are associated with a variety of genetic alterations and the aberrant reactivation of developmental signaling pathways, such as hyaluronan synthase2 [13], focal adhesion kinase-related non-kinase [14], and phosphatase and tensin homologue deleted on chromosome 10 (PTEN) [15–17].

USP13 has been recognized as a deubiquitylase that reverses PTEN polyubiquitylation and stabilizes PTEN protein. Zhang and colleagues have demonstrated that USP13 expression was reduced in human breast tumors and that USP13 depletion could promote tumorigenesis through the down-regulation of PTEN [18]. IPF is also considered to be a neoproliferative lung disorder due to its similarity to tumors [19]. However, little is known about the role of USP13 in IPF. PTEN, as a tumor suppressor, is involved in multiple cellular processes, such as cell survival, apoptosis, adhesion, and cell migration/invasion [20]. The loss of PTEN expression has been reported to correlate with fibroproliferation in IPF by White and colleagues [17]. Additionally, numerous studies have confirmed that PTEN expression is decreased in fibroblasts isolated from IPF patients, which could account for the increased proliferative and migratory/invasive capacities of IPF fibroblasts [15–17]. These findings prompted us to explore the relationship between USP13 and PTEN in IPF pathogenesis.

In this study, our microarray assay revealed that USP13 mRNA expression was decreased in IPF lung tissues. Moreover, we also observed diminished USP13 expression in the fibroblastic foci of usual interstitial pneumonia (UIP) lungs and in the primary lung fibroblasts isolated from IPF patients. We noticed that a USP13 deficiency led to the transformation of fibroblasts into an aggressive phenotype with enhanced proliferative, migratory, and invasive capacities. Additionally, we found a correlation between USP13 loss and reduced PTEN expression in IPF fibroblasts. Hence, our data suggest that USP13, as a modulator regulating PTEN expression, is decreased in IPF fibroblasts and thereby contributes to phenotypic changes in fibroblasts, which might be essential events in IPF pathogenesis.

## Materials and methods

### Subjects and cells

Thirteen IPF patients with UIP confirmed by a surgical lung biopsy were included in this study. All patients were males with an average age of 51.08 ± 10.03 years. The diagnosis of IPF was made according to the standards accepted by ATS/ERS/JRS/ALAT [1]. Five primary IPF fibroblast lines were established from fresh lung tissues obtained from five patients, who were a subset of the 13 patients described above. Three primary normal fibroblast lines were isolated from histologically proven normal lung tissue from patients with primary spontaneous pneumothorax. Five normal, formalin-fixed, paraffin-embedded (FFPE) lung specimens from archived tumor-adjacent healthy lung parenchyma of patients with non-small cell lung cancer were used for immunohistochemistry. One normal human fetal pulmonary fibroblast cell line (MRC-5) was purchased from American Type Culture Collection (ATCC, Manassas, VA, USA). This study was approved by the Ethics Committee of Beijing Chao-Yang Hospital of Capital Medical University, Beijing, China, and written informed consent was obtained according to institutional guidelines from all investigated subjects.

Primary fibroblast cell lines were generated by explant culture and cultured in high glucose Dulbecco’s modified Eagle’s medium (DMEM, HyClone, Logan, UT, USA) containing 10 % fetal bovine serum (FBS, Invitrogen, Carlsbad, CA, USA), 100 U/ml penicillin (Hyclone), and 100 mg/ml streptomycin (Hyclone), as previously described [21]. Primary cells were characterized as fibroblasts, as shown in our previous reports [22, 23]. MRC-5 were maintained in Minimum Essential Medium (MEM)-α (Invitrogen) with 10 % FBS (Invitrogen), 100 U/ml penicillin (Hyclone), and 100 mg/ml streptomycin (Hyclone).

Under normoxic conditions, MRC-5 cells were cultured in a humidified incubator (Thermo Scientific, Waltham, MA, USA) at 37 °C in 95 % air (21 % O_2_) and 5 % CO_2_. A Heracell incubator (Thermo Scientific) filled with nitrogen gas was used for hypoxic conditions (94 % N_2_, 5 % CO_2_, and 1 % O_2_). All cell lines in this study were used between passages five and eight.

### Microarray experiment

The lung tissues that were used as the source of our primary fibroblast lines were also used for our microarray experiment. They were obtained from five IPF patients and three normal control subjects. Gene expression profiling was performed according to the manufacturer’s instructions using the GeneChip Human Transcriptome Array 2.0 (Affymetrix, California, USA), which provided probes for approximately 44,699 genes from the human genome. Total RNA was isolated with TRIzol reagent (Invitrogen) and purified using a RNeasy Mini Kit (Qiagen, Hilden, Germany). The RNA concentration was determined using a NanoDrop 2000 (Thermo Scientific). The RNA purity and quality was assessed by its A260/A280 ratio and agarose gel electrophoresis (Additional file 1: Figure S1). Biotinylated cDNA were prepared from 250 ng total RNA by using an Ambion WT Expression Kit (Affymetrix) according to the manufacturer’s instructions. After labeling, 5.5 μg cDNA was hybridized to the GeneChip Human Transcriptome Array 2.0. GeneChips were washed and stained in a GeneChip Fluidics Station 450 (Affymetrix). GeneChips were then scanned by using the Affymetrix GeneChip Command Console Software that was installed in GeneChip Scanner 3000 7G (Affymetrix). Raw data were normalized at the transcript level using the Robust Multi-Chip Average (RMA) method [24]. A random variance model (RVM) [25] *t*-test was applied to filter the differentially-expressed genes for the control and experiment groups. A *p* value <0.05 was considered to indicate a significant difference. Complete microarray data were deposited in the Gene Expression Omnibus website (Accession number GSE72073).

### Immunohistochemistry

Immunohistochemistry staining was performed on 4-mm sections of FFPE lung specimens from 13 IPF patients and five normal controls. Paraffinized sections were deparaffinized in xylene and rehydrated through a series of alcohol to water. Slides were stained with hematoxylin and eosin (H&E) and viewed under a light microscope. For immunohistochemistry, the tissue sections were deparaffinized and rehydrated as described above. After a microwave treatment for 20 min in ethylenediaminetetraacetic acid (EDTA) buffer and subsequent cooling, the endogenous peroxidase activity was blocked by 0.3 % hydrogen peroxide in methanol for 15 min. After three washes followed by blocking in 5 % skim milk in phosphate-buffered saline (PBS) for 30 min, sections were incubated with antibodies against PTEN (clone 6H2.1, Cascade Bioscience, Winchester, MA, USA), USP13 (Abcam, Cambridge, MA, USA), and α-SMA (R&D, Minneapolis, MN, USA) overnight at 4 °C. Sections were incubated with horseradish peroxidase-conjugated secondary antibodies and visualized using a 3, 3'-diaminobenzidine (DAB) kit (Maixin Biotech, Fuzhou, China). All slides were scanned using a Aperio ScanScope® AT (Leica, Nussloch, Germany), and staining of USP13 was analyzed with Aperio software.

### Small interfering RNA (siRNA) transfection

MRC-5 cells were seeded in 6-well plates and incubated overnight. USP13 siRNA (5 μM) and negative control (NC) siRNA (5 μM) were separately mixed with RNAiMAX transfection reagent (Invitrogen) according to the manufacturer’s instructions. siRNA–RNAiMAX complexes were added to MRC-5 cells following two washes with serum-free medium. After 24 h, the transfection medium was replaced by culture medium for another 24 h. A similar method with PTEN siRNA was used for PTEN siRNA transfection. USP13 siRNA #1 (s17129), USP13 siRNA #2 (s17130), PTEN siRNA (s325), and negative control siRNA were all Silence® Select siRNAs and were purchased from Invitrogen (Carlsbad, CA, USA).

### RNA purification, reverse transcription, and real-time RT-PCR analysis

Total RNA was isolated using the TRIzol reagent (Invitrogen) according to the manufacturer’s instructions. Reverse transcription was performed on 2 μg total RNA with oligo (dT) primers in 25 μl reactions using the Omniscript RT kit (Tiangen Biotech, Beijing, China) according to the manufacturer’s instructions. Real-time RT-PCR was carried out on an ABI PRISM® 7500 instrument (Applied Biosystems, Foster, CA, USA) using SYBR-Green PCR reagents (Tiangen Biotech) as previously described [22]. The primers used were USP13-F: 5′-CCTGATGAACCAATTGATAGACC-3′; USP13-R: 5′-GTGATGATAGCTACGATTTCCTC-3′, and PTEN-F: 5′-CGGCAGCAAATGTTTCAG-3′; PTEN-R: 5′-AACTGGCAGGTAGAAGGCAACTC-3′. For each sample, β-actin was used for normalization (β-actin-F: 5′-TGCTATCCAGGCTGTGCTAT-3′; β-actin-R: 5′-AGTCCATCACGATGCCAGT-3′). The fold-change of the target genes was calculated by using the 2^-∆∆CT^ method.

### Protein extraction and western blot analysis

Total cell lysates were obtained using RIPA buffer (Solarbio, Beijing, China) containing 1:100 phenylmethylsulfonyl fluoride and phosphatase inhibitors. Cell lysates were resuspended in protein loading buffer containing 5 % mercaptoethanol. The proteins were separated by 10 % sodium dodecyl sulfate polyacrylamide gel electrophoresis (SDS-PAGE) (Bio-Rad, Hercules, CA, USA) using a Mini-Protean electrophoresis module assembly (Bio-Rad) at 80 mV and transferred to nitrocellulose membranes (Millipore, Billerica, MA, USA) for 100 min using the Mini Trans-Blot electrophoresis transfer cell (Bio-Rad) at 300 mA. The membranes were treated with IRDyeTM800 (green)- or IRDyeTM700 (red)-conjugated affinity purified anti-rabbit or anti-mouse IgG (LI-COR, Lincoln, NE, USA). Positive bands were visualized, and the intensity of the bands was evaluated using a LI-COR Odyssey infrareddouble-fluorescence imaging system (LI-COR). The primary antibodies used were anti-human USP13 (ab99421, Abcam), PTEN (ab32199, Abcam), and β-actin (CB100997M, California Bioscience, Coachella, CA, USA).

### Immunofluorescence staining

MRC-5 cells seeded on glass slides were fixed in 90 % cold ethanol, permeabilized by 0.1 % Triton X100 in PBS, blocked with 5 % bovine serum albumin (BSA), and incubated with anti-PTEN (26H9, #9556, Cell Signaling Technology, Danvers, MA, USA) or anti-USP13 (ab99421, Abcam) overnight at 4 °C. Cells were washed and incubated with Alexa Fluor® 594-conjugated goat anti-mouse/488-conjugated goat anti-rabbit secondary antibodies (Jackson ImmunoResearch, West Grove, PA, USA) for 50 min in the dark. Cells were washed three times, mounted with 4'6-diamidino-2-phenylindole (DAPI)-containing mounting media (ZSGB-BIO, Beijing, China), and visualized using a confocal laser scanning microscope (Leica Microsystems, Wetzlar, Hesse, Germany).

### Bromodeoxyuridine (BrdU) cell proliferation assay

MRC-5 cells were seeded on glass slides in 24-well plates and transfected with PTEN siRNA or NC siRNA for 24 h without FBS, followed by incubation with MEM-α containing 10 % FBS and antibodies for another 24 h. BrdU incorporation was measured using a BrdU labeling and detection kit (Roche, Mannheim, Germany) in accordance with the manufacturer’s protocol. Briefly, MRC-5 cells were labeled with 10 μM BrdU during an incubation of 50 min. After labeling, the cells were washed three times, fixed, and treated with mouse anti-BrdU monoclonal antibody for 30 min at 37 °C, followed by an anti-mouse-Ig-fluorescein working solution. Cells were washed three times and mounted with DAPI-containing mounting media (ZSGB-BIO, Beijing, China). Cellular proliferation was assessed by the percent of BrdU-staining cells in nuclear-positive cells in the same microscopic visual field. Experiments were performed three independent times. The BrdU positive rate was evaluated by counting five different microscopic fields per time point.

### Migration/invasion assay

Migration assays were performed using 8-μm pore transwell chambers (Millipore). In invasion assays, transwell chambers with 8-μm pores were coated with 30 μl of matrigel (BD Biosciences, San Diego, CA, USA) gel mixture (1:5 in MEM-α containing 0.1 % FBS), and allowed to solidify for 1 h at 37 °C.

Briefly, cells were transfected with PTEN siRNA or NC siRNA for 24 h prior to detachment with 0.125 % trypsin (Hyclone). A total of 2 × 10^4^ cells were resuspended in MEM-α media containing 0.1 % FBS and added to the upper chamber. MEM-α media containing 10 % FBS was added to the lower chamber. Cell viability was assessed by crystal violet staining and microscopic evaluation.

### Immunoprecipitation

Whole cell lysates were obtained using lysis buffer (50 mM HEPES at pH 8.0, 150 mM NaCl, 0.5 % Triton X-100, 0.5 % NP-40, 1 mM DTT, cocktail inhibiter, and 10 % glycerol) and mixed with pre-cleared protein A/G agarose beads (Santa Cruz Biotechnology, CA, USA) for 2 h. The lysates were then immunoprecipitated with the indicated antibodies and protein A/G beads overnight at 4 °C. Beads were washed four times with lysis buffer and boiled in 2× loading buffer. Protein samples were resolved by 8 % SDS-PAGE (Bio-Rad, Hercules, CA, USA) and transferred onto a polyvinylidene difluoride membrane (Millipore, Billerica, MA, USA), and membranes were probed with the indicated antibodies. The primary antibodies used were human USP13 (ab99421, Abcam) and PTEN (26H9, #9556, Cell Signaling Technology). Reactive proteins were detected by the ECL Chemiluminescence System (Millipore).

### Ubiquitination assay

Cells were transfected with USP13 siRNA and treated with 10 μM proteasome inhibitor (MG-132, Millipore) for 6 h before harvest or stimulated with hypoxia for 48 h, as indicated. Whole cells were lysed in lysis buffer (50 mM Tris–HCl at pH 8.0, 150 mM NaCl, 5 mM DTT, and 10 % glycerol). After boiling for 5 min, lysates were diluted 100-fold with cold lysis buffer containing an inhibitor cocktail. After immunoprecipitation with the indicated antibodies, the immunoprecipitates were resolved by 8 % SDS-PAGE (Bio-Rad) and transferred onto a polyvinylidene difluoride membrane (Millipore). The blot was blocked in 5 % BSA and probed with an anti-ubiquitin antibody (ab7780, Abcam) according to the manufacturer’s instructions. Reactive proteins were detected by the ECL Chemiluminescence System (Millipore).

### Statistical analysis

Data are expressed as the mean ± SEM where applicable. We assessed differences in measured variables using the unpaired/paired two-sided Student’s *t-*test or Mann–Whitney test for nonparametric data. *p* <0.05 was considered statistically significant. SPSS 16.0 (SPSS Inc., Chicago, IL, USA) and GraphPad Prism 5.0 (Graphpad Software, La Jolla, CA, USA) were used for statistical analysis and graph creation.

## Results

### Decreased USP13 expression in tissues from IPF patients

**Fig. 1:**
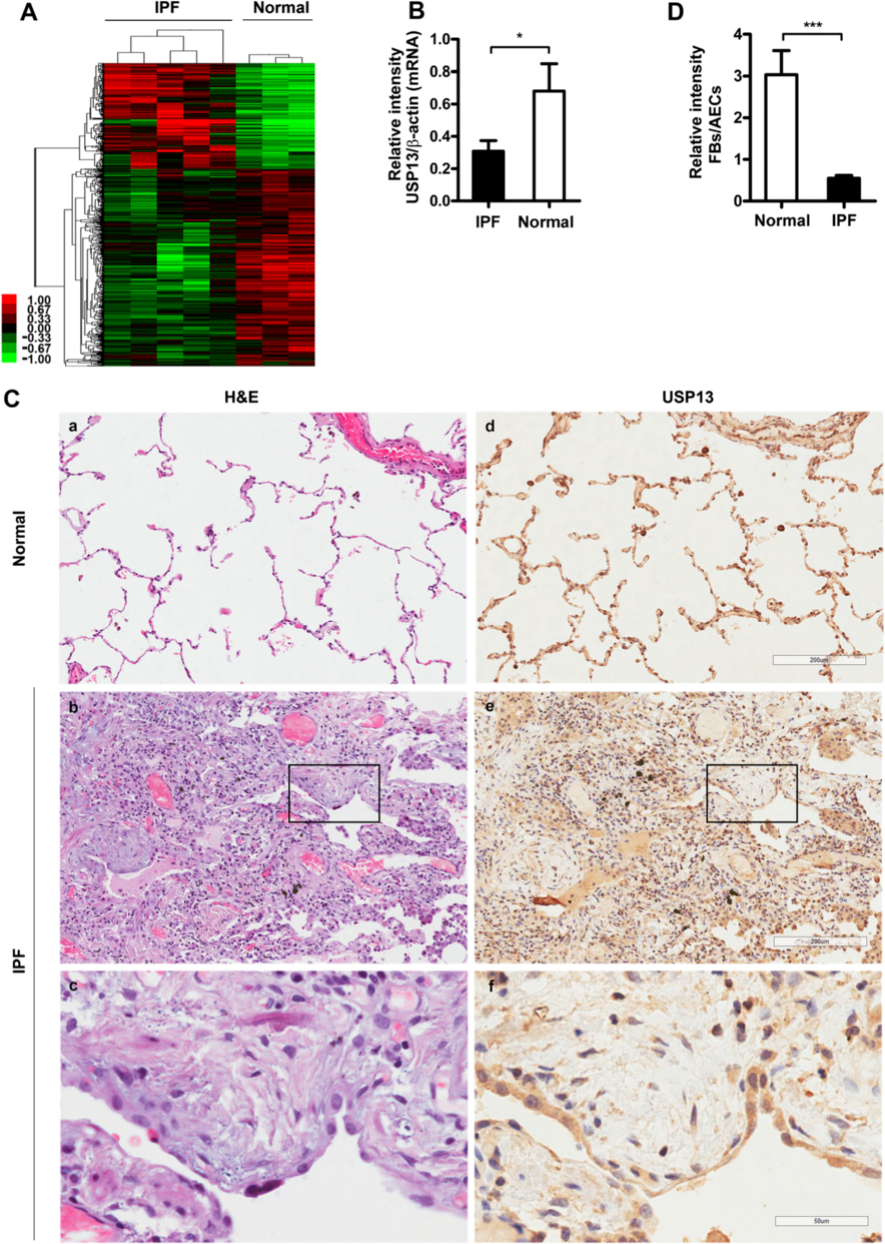
USP13 expression levels in IPF lung tissues. **a** Gene expression profiles of fibrotic lung tissue from IPF patients (*n* = 5) and of normal lung tissues from primary spontaneous pneumothorax patients (*n* = 3). Red areas indicate increased gene expression relative to the mean of all samples, and green areas indicate decreased gene expression. Each *column* represents one patient, and each *row* represents one gene. **b** Graph of USP13 mRNA levels in representative samples. USP13 mRNA expression levels of representative samples were verified by real-time RT-PCR. β-actin was used as a control. **c** USP13 expression in IPF lung tissue fibroblastic foci. Representative serial lung sections from one patient with IPF and one normal control were stained for H&E (**a**, **b**, **c**) and USP13 (**d**, **e**, **f**). A fibrotic focus is indicated with a black box in (**b**) and (**e**). High magnification of the boxed region in (**c**) and (**f**). (**a**, **b**, **d**, **e**) original magnification × 100; (**c**, **f**) original magnification × 400. **d** Relative intensity analysis of USP13 staining in IPF lung tissue fibroblastic foci (*n* = 13) and normal control (*n* = 5). The quantitative data were expressed as the ratio of stained areas in myofibroblasts within FF (IPF) or mesenchymal cells (Normal) to the overlying epithelial cells. Staining intensity was represented by the percentage of positive-staining cells in the indicated tissues. Error bars indicate the mean ± SEM. *p <0.05, ***p <0.001

**Fig. 2:**
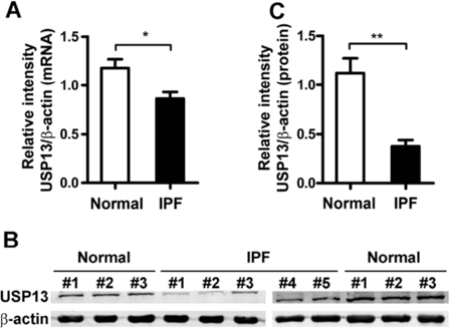
USP13 expression levels in primary fibroblast lines from IPF patients. **a** Graph of USP13 mRNA expression in primary IPF fibroblast lines (*n* = 5) and primary normal fibroblast lines (*n* = 3). USP13 mRNA expression levels were verified by real-time RT-PCR. **b** Protein expression levels of USP13 in the primary fibroblast lines were shown by western blot. **c** Graph of USP13 protein levels in representative primary fibroblast lines was shown. Relative intensity analysis of USP13 protein expression levels were assessed by fold-change of target protein/β-actin with standardization by setting the “normal control #1” as 1. β-actin was used as a control in both western blot and real-time RT-PCR assays. Error bars indicate the mean ± SEM. *p <0.05, **p <0.01

To identify gene expression profiles involved in the pathogenesis of IPF, we performed a comparative cDNA microarray analysis of the lung tissues from IPF patients (*n* = 5) and control subjects (*n* = 3). We found 1476 differentially-expressed genes between the two groups of patients at a significance threshold of *p* <0.05 (Fig. 1a). Given the known role of USP13 in the deubiquitylation and stabilization of PTEN, we were particularly interested in the down-regulation of USP13 in IPF (Fold-change = 0.78 and *p* = 0.017 in microarray data, real-time RT-PCR validation in Fig. 1b). To further evaluate USP13 expression in IPF, we first performed immunohistochemistry on surgical lung biopsy specimens from 13 IPF patients with a pathologic diagnosis of UIP. In 11 out of 13 IPF patients, USP13 staining was diminished or absent in fibroblastic foci compared with that in the overlying epithelial layers (Fig. 1c and d). We then examined USP13 expression in primary human lung fibroblast lines isolated from fresh lung tissues of five IPF patients and three normal lung tissues. As shown in Fig. 2, USP13 protein and mRNA levels were visibly decreased in primary IPF fibroblast lines compared with those in primary normal fibroblast lines.

### USP13-deficient fibroblasts display an aggressive phenotype

In view of the striking difference in the level of USP13 expression between IPF and normal lung fibroblasts, we investigated the effects of USP13 loss on the phenotype of fibroblasts, specifically their proliferative capacity and migratory/invasive capacity.

**Fig. 3:**
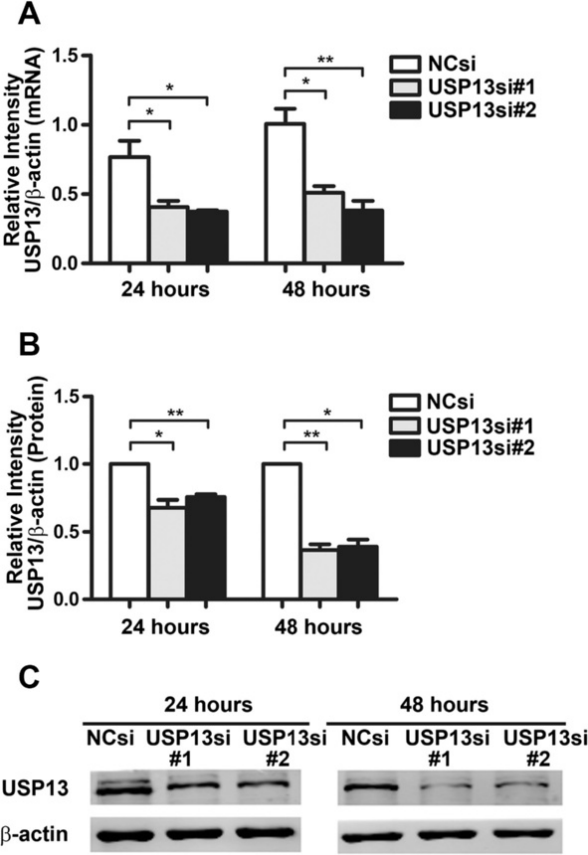
Knockdown of USP13 through RNA interference. MRC-5 cells were transfected with negative control siRNA (NCsi) or two different USP13 siRNAs (USP13si #1 and #2) for 24 h or 48 h. **a** Graph of USP13 mRNA levels at both 24 h and 48 h in USP13 siRNA-treated cells. **b** Graph of USP13 protein levels at 24 h or 48 h after USP13 siRNA transfection. **c** Protein expression levels of representative samples are shown. Error bars indicate the mean ± SEM. *p <0.05, **p <0.01

We knocked down USP13 expression in normal lung fibroblasts (MRC-5 cells) by using siRNA interference. As shown in Fig. 3, two USP13 siRNA (USP3si #1 and USP13si #2, 5 μM) were tested and both of them significantly down-regulated USP13 mRNA and protein expression levels at 48 h post-transfection, compared with the negative control siRNA (NCsi). Therefore, either of them could be used in the subsequent experiments, MRC-5 cells were treated with a siRNA against USP13 or a negative control for 48 h, respectively named MRC-5^USP13si^ cells and MRC-5^NCsi^ cells.

**Fig. 4:**
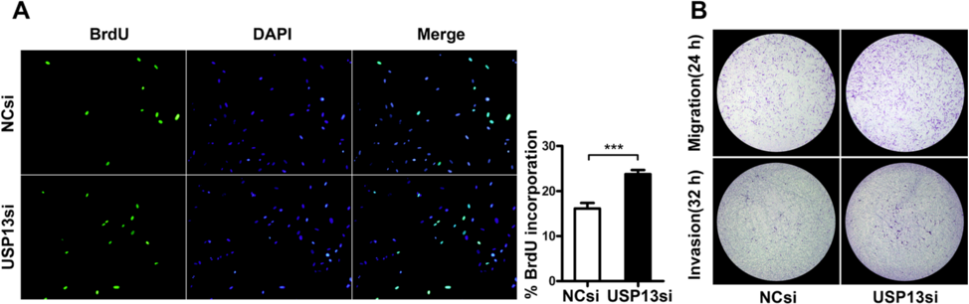
Proliferative, migratory, and invasive capacity of fibroblasts following the down-regulation of USP13. **a** Proliferation of cells with USP13 deficiency. At 48 h after transfection with USP13 siRNA (USP13si) or NC siRNA (NCsi), MRC-5 cell proliferation was assessed by a BrdU incorporation assay. The proliferating cells that recently underwent DNA synthesis were labeled with BrdU (green), and total nuclei were visualized by DAPI (blue) staining. % BrdU: Percent of BrdU^+^ cells within the siRNA-treated MRC-5 cell population. Error bars indicate the mean ± SEM. ***p <0.001. **b** The migratory and invasive capacity of fibroblasts with decreased USP13. Twenty-four hours after transfection with either USP13 siRNA or NC siRNA, equal numbers of fibroblasts were loaded into transwell chambers with or without matrigel. Images of migratory fibroblasts (top panel) and invasive fibroblasts (bottom panel) are shown from a crystal violet staining assay at different time points, as indicated

We next measured cell proliferation using BrdU incorporation assays in MRC-5^USP13si^ cells and MRC-5^NCsi^ cells. As shown in Fig. 4a, USP13 deficiency significantly increased fibroblast proliferation.

We then assessed the effects of USP13 reduction on the migratory and invasive capacities of fibroblasts. After transfection with USP13 siRNA or NC siRNA for 24 h, equal numbers of fibroblasts were loaded into a transwell chamber with or without matrigel. USP13 deficiency also dramatically promoted the invasive and migratory capacity of normal lung fibroblasts *in vitro* (Fig. 4b).

### USP13 deficiency accounts for the decreased PTEN protein expression in IPF fibroblasts

Considering that loss of PTEN contributed to fibroblasts phenotypic changes and USP13 interacted with PTEN and functioned as a deubiquitylase in epithelial cells, we subsequently sought to determine whether USP13 deficiency is responsible for the low PTEN expression observed in IPF fibroblasts.

**Fig. 5:**
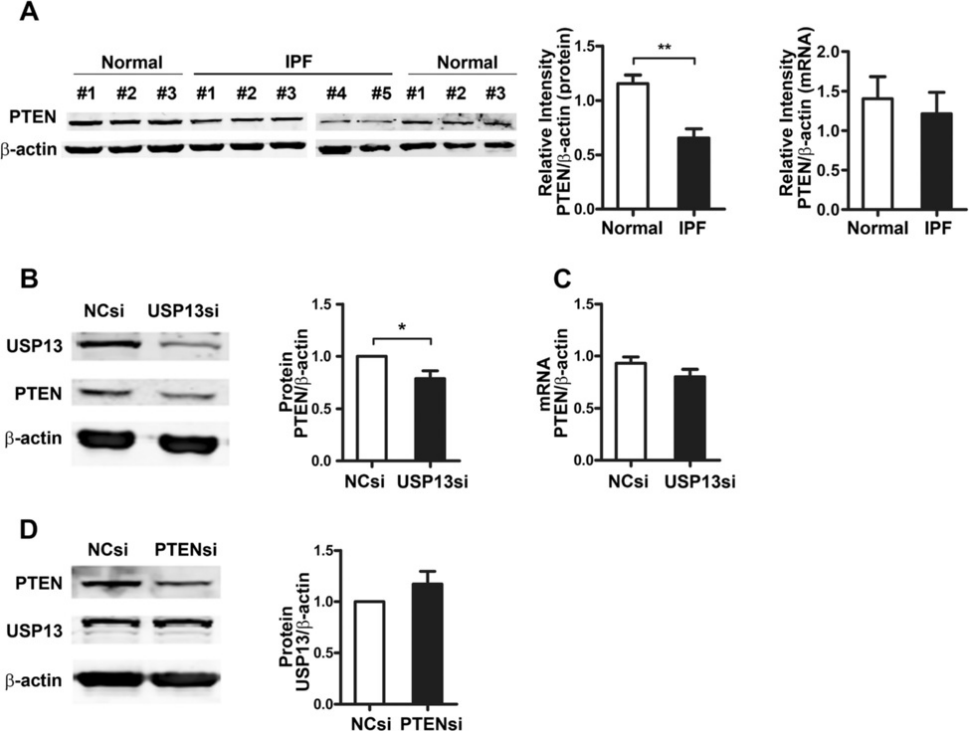
The PTEN protein level in UPS13-deficient cells. **a** PTEN protein and mRNA levels in primary IPF fibroblasts. Relative intensity analysis of PTEN protein expression levels in primary IPF fibroblast lines (*n* = 5) and primary normal fibroblast lines (*n* = 3) were assessed by the fold-change of target protein/β-actin with standardization by setting the “normal control #1” as 1. PTEN mRNA expression was measured by real-time RT-PCR, and the fold-change was calculated by using the 2^-∆∆CT^ method. **b**, **c** MRC-5 cells were transfected with USP13 siRNA (USP13si) or NC siRNA (NCsi) for 48 h and PTEN protein (**b**) and mRNA (**c**) levels were measured by western blot and real-time RT-PCR. **d** MRC-5 cells were transfected with PTEN siRNA (PTENsi) or NCsi for 48 h, and USP13 protein expression was assessed by western blot. β-actin was used as a control in both the western blot and real-time RT-PCR assays. Error bars indicate the mean ± SEM. *p <0.05, **p <0.01

Consistent with previous reports, we discovered that PTEN protein expression was markedly reduced in the IPF fibroblast lines, compared with that in normal fibroblasts, but no significant difference in the level of PTEN mRNA expression was found between these two groups (Fig. 5a).

We then examined the effects of USP13 deficiency on PTEN protein and mRNA levels. As shown in Fig. 5b and c, compared with MRC-5^NCsi^ cells, MRC-5^USP13si^ displayed a marked decrease in PTEN protein levels with no obvious change in their mRNA levels. Additionally, our data showed that the suppression of PTEN by siRNA did not lead to a reduction in USP13 expression (Fig. 5d). These data suggest that USP13 regulates the PTEN protein expression level, but PTEN does not affect USP13 expression.

**Fig. 6:**
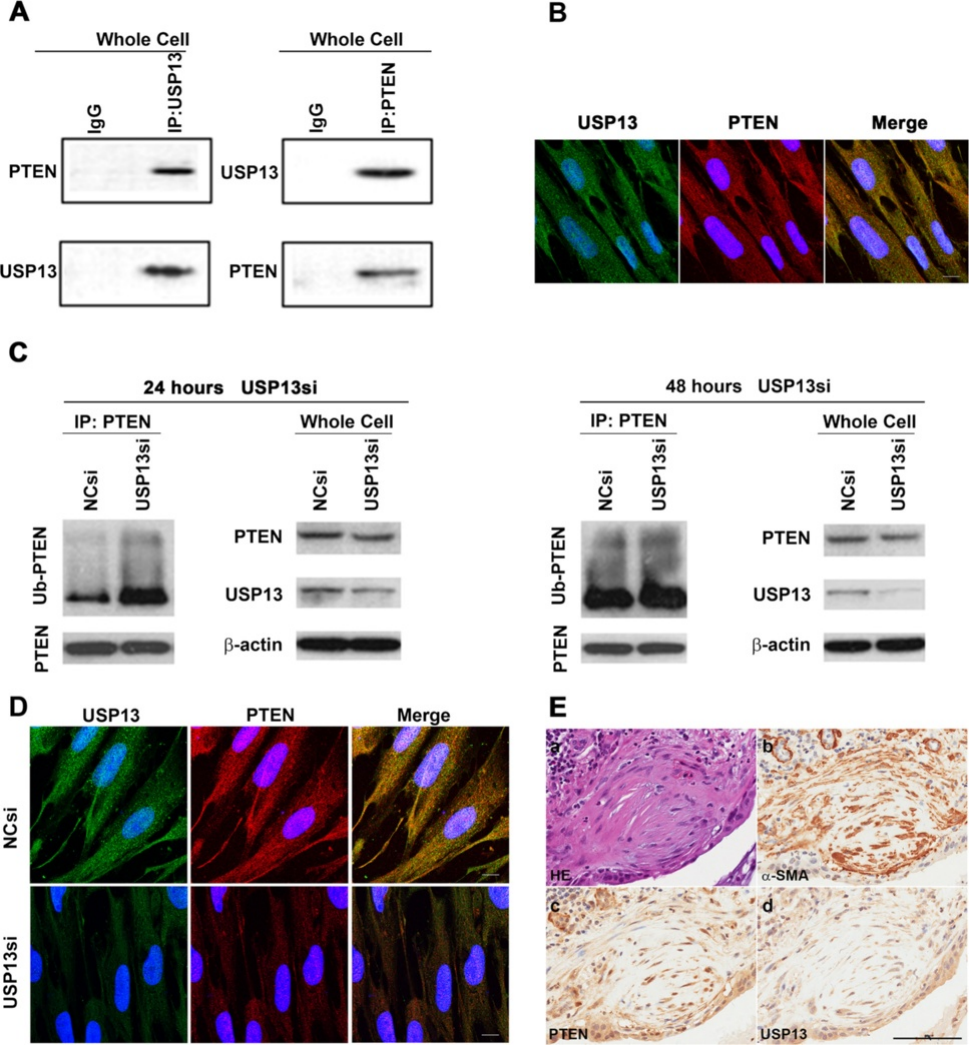
Levels of PTEN ubiquitination and degradation in USP13-deficient lung fibroblasts. **a** Endogenous USP13 was immunoprecipitated from MRC-5 whole cell lysates and immunoblotted with antibodies against USP13 and PTEN (left panel), while endogenous PTEN was immunoprecipitated from MRC-5 whole cell lysates and immunoblotted with antibodies against PTEN and USP13 (right panel). **b** Co-localization of USP13 and PTEN. PTEN (red) and USP13 (green) were labeled by immunofluorescence staining of MRC-5 cells. The right panel shows the overlay (merge) of PTEN, USP13, and nuclear DAPI staining (blue) in the same field to determine co-localization. Scale bar, 20 μm. **c** At 42 h after transfection with USP13 siRNA or NC siRNA, we added MG-132 (10 μM) for 6 h and harvested whole cell lysates. PTEN and ubiquitinated PTEN (Ub-PTEN) were assessed by immunoprecipitation and western blot. β-actin was used as a loading control. **d** Immunofluorescence staining of PTEN (red) and USP13 (green) in MRC-5 cells transfected with USP13 siRNA (USP13si) or negative control siRNA (NCsi) for 48 h. Nuclei were visualized by DAPI (blue) staining. Scale bar, 20 μm. (**a**–**d**) Data are representative of at least three independent experiments. **e** Representative serial lung sections from one IPF patient were stained for (**a**) H&E, (**b**) α-SMA, (**c**) PTEN, and (**d**) USP13. Images are representative of staining performed on samples from 11 individuals with IPF. Scale bar, 100 μm

Next, we performed a co-immunoprecipitation with whole cell lysates from untreated MRC-5 cells and found an association of PTEN with USP13 (Fig. 6a). To further validate the interaction between USP13 and PTEN, an immunofluorescent confocal microscopic analysis was conducted. Our results (Fig. 6b) showed a co-localization of USP13 and PTEN in lung fibroblasts. Additionally, we noticed that in fibroblasts, the loss of USP13 down-regulated PTEN protein expression by increasing PTEN ubiquitination. As shown in Fig. 6c, compared with MRC-5^NCsi^ cells, in whole cell lysates of MRC-5^USP13si^ cells, the expression of ubiquitinated PTEN was enhanced while that of total PTEN protein was decreased.

To further confirm the effects of USP13 loss on PTEN expression, we performed immunocellularchemical staining of USP13 and PTEN in MRC-5^USP13si^ cells and MRC-5^NCsi^ cells. We observed that USP13 deficiency led to a marked reduction in the level of PTEN protein expression (Fig. 6d).

Additionally, we performed immunohistochemical staining of PTEN in the lung tissue samples of 13 IPF patients to further validate the correlation between USP13 loss and reduced PTEN expression in IPF fibroblasts. In the same 11 IPF patients, we observed a weaker staining of both USP13 and PTEN and a stronger staining of α-SMA in myofibroblasts within fibroblastic foci, compared with those in the overlying epithelial cells (Fig. 6e).

Collectively, these data revealed a strong correlation between USP13 deficiency and down-regulated PTEN expression in IPF fibroblasts.

### Hypoxia-reduced expression of USP13 correlates with PTEN protein loss

**Fig. 7:**
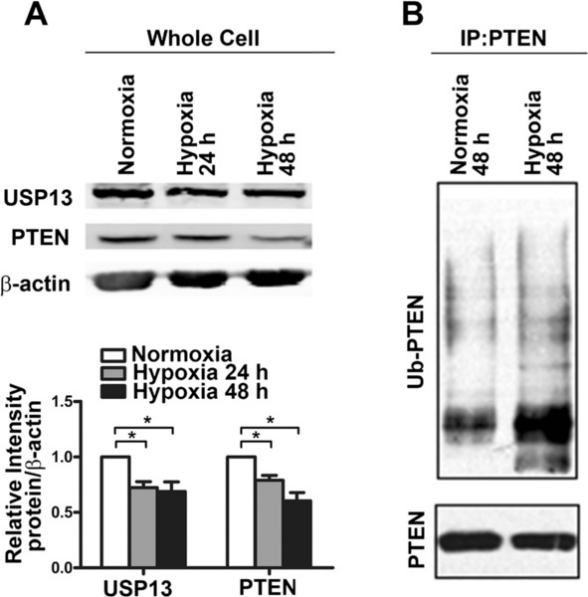
Levels of USP13 and PTEN during hypoxia, **a** Expression of USP13 and PTEN in a hypoxic environment. MRC-5 cells were exposed to normoxic (21 % O_2_) or hypoxic (1 % O_2_) conditions for 24 h or 48 h. Error bars indicate the mean ± SEM. *p <0.05. **b** PTEN ubiquitination in a hypoxic environment. MRC-5 cells were exposed to normoxic (21 % O_2_) or hypoxic (1 % O_2_) conditions for 48 h, and then PTEN and ubiquitinated PTEN (Ub-PTEN) were assessed by immunoprecipitation and western blot. Data are representative of at least three independent experiments

Hypoxia has been recognized as a prominent microenvironmental factor in tissue injury, normal wound healing, and fibrosis [26]. We exposed MRC-5 cells to normoxic (21 % O_2_) or hypoxic (1 % O_2_) conditions for 48 h and discovered that hypoxia induced a significant decrease in the level of USP13 expression, coinciding with a reduction in the level of PTEN protein expression, as well as an increase in the level of PTEN ubiquitination (Fig. 7). These findings strongly suggest that under hypoxic conditions, USP13 down-regulation and low PTEN expression exist in lung fibroblasts.

## Discussion

It is generally accepted that IPF involves an aberrant would healing in response to repetitive lung injury, and ultimately results in fibrosis [3]. The fibroblast is considered to be the classical cell type involved in the pathogenesis of IPF and the major contributor to the pool of myofibroblasts in fibroblastic foci [10]. A prerequisite for this consideration is the migration of IPF fibroblasts to the site of injury and their subsequent proliferation and transformation [27]. In this study, we found diminished USP13 mainly in IPF fibroblasts, which contributed to fibroblast migration, invasion, and proliferation. Our observation suggests that USP13, serving as an important regulator of fibroblast biologic behaviors, plays a critical role in the pathogenesis of IPF.

Recently, mounting evidence supports the existence of similarities between IPF and cancer in both pathogenesis and biology [19, 28, 29]. The critical role of PTEN in the pathogenesis of IPF has drawn attention due to its role as a tumor suppressor [16, 30]. PTEN protein, as a dual-specificity lipid and protein phosphatase, antagonizes the phosphoinositide 3-kinase (PI3K)-Akt signal pathway and dephosphorylates focal adhesion kinase (FAK), which has been implicated in carcinogenesis and in the pathogenesis of IPF [17, 31, 32]. It has been shown that decreased PTEN expression or activity is associated with pathological phenotypes of IPF fibroblasts [17, 33]. Xia has demonstrated that low caveolin-1 expression led to diminished membrane PTEN levels and low PTEN phosphatase activity in IPF fibroblasts [15]. However, the mechanisms responsible for this decreased PTEN expression in IPF fibroblasts have not been well established. Our data show that PTEN protein, but not PTEN mRNA, levels were decreased in IPF fibroblasts, strongly implying that PTEN down-regulation is due to posttranslational modification. It was reported that USP13 acted as a deubiquitylase of PTEN in breast cancer [18]. Interestingly, we found that USP13 interacted with PTEN in fibroblasts and regulated PTEN ubiquitination and degradation, supporting the idea that IPF is a cancer-like disease. Consistently, both USP13 and PTEN expression were diminished in myofibroblasts within fibroblastic foci in 11 out of 13 (84.6 %) IPF patients. These data confirm a strong correlation between USP13 and PTEN in IPF. As a deubiquitylase, USP13 has been reported to cooperate with gp87 to promote endoplasmic reticulum (ER)-associated degradation (ERAD) [34] and to interact with Siah2, subsequently regulating its activity [35]. Hence, it is possible that USP13 contributes to the pathogenesis of IPF through different pathways. Further studies are needed to explore this possibility.

Hypoxia is an important factor in various lung pathological processes, including pulmonary fibrosis [36]. Hypoxia has been shown to enhance fibroblast proliferation with an increased production of extracellular matrix [37, 38]. Our data demonstrate that when exposed to hypoxia, the expression levels of both USP13 and PTEN were down-regulated in fibroblasts. This finding points to a new regulatory mechanism of fibroblast phenotypes under hypoxic conditions. Furthermore, the finding of a diminished level of USP13 concurrent with a low PTEN protein level in myofibroblasts within fibroblastic foci or in fibroblasts maintained under hypoxia implies that the loss of USP13 is a key mechanism responsible for PTEN down-regulation in IPF fibroblasts.

In conclusion, USP13 was down-regulated in lung tissues from patients with IPF, especially in fibrotic foci, and in primary IPF fibroblast lines. USP13 deficiency, via down-regulating the PTEN protein level and subsequently mediating the phenotypic changes of lung fibroblasts, contributes to the pathogenesis of IPF. Thus, USP13 may be a novel potential target for intervention in lung fibrosis.

## Additional file

Additional file 1: Figure S1. The RNA purity and quality analysis. (A) The A260/A280 ratio of RNAs isolated from lung tissues of five IPF patients and three normal control subjects. (B) The representative image of the same RNAs showed by agarose gel electrophoresis. (PDF 903 kb)

## References

[1] Raghu G, Collard HR, Egan JJ, Martinez FJ, Behr J, Brown KK (2011). An official ATS/ERS/JRS/ALAT statement: idiopathic pulmonary fibrosis: evidence-based guidelines for diagnosis and management. Am J Respir Crit Care Med.

[2] Cai M, Zhu M, Ban C, Su J, Ye Q, Liu Y (2014). Clinical features and outcomes of 210 patients with idiopathic pulmonary fibrosis. Chin Med J (Engl).

[3] Selman M, King TE, Pardo A, American Thoracic S, European Respiratory S, American College of Chest P (2001). Idiopathic pulmonary fibrosis: prevailing and evolving hypotheses about its pathogenesis and implications for therapy. Ann Intern Med.

[4] Uhal BD, Joshi I, Hughes WF, Ramos C, Pardo A, Selman M (1998). Alveolar epithelial cell death adjacent to underlying myofibroblasts in advanced fibrotic human lung. Am J Physiol.

[5] Nicholson AG, Fulford LG, Colby TV, du Bois RM, Hansell DM, Wells AU (2002). The relationship between individual histologic features and disease progression in idiopathic pulmonary fibrosis. Am J Respir Crit Care Med.

[6] Tiitto L, Bloigu R, Heiskanen U, Paakko P, Kinnula VL, Kaarteenaho-Wiik R (2006). Relationship between histopathological features and the course of idiopathic pulmonary fibrosis/usual interstitial pneumonia. Thorax.

[7] Visscher DW, Myers JL (2006). Histologic spectrum of idiopathic interstitial pneumonias. Proc Am Thorac Soc.

[8] Maher TM, Wells AU, Laurent GJ (2007). Idiopathic pulmonary fibrosis: multiple causes and multiple mechanisms?. Eur Respir J.

[9] Thannickal VJ, Horowitz JC (2006). Evolving concepts of apoptosis in idiopathic pulmonary fibrosis. Proc Am Thorac Soc.

[10] Hung C, Linn G, Chow YH, Kobayashi A, Mittelsteadt K, Altemeier WA (2013). Role of lung pericytes and resident fibroblasts in the pathogenesis of pulmonary fibrosis. Am J Respir Crit Care Med.

[11] Ramos C, Montano M, Garcia-Alvarez J, Ruiz V, Uhal BD, Selman M (2001). Fibroblasts from idiopathic pulmonary fibrosis and normal lungs differ in growth rate, apoptosis, and tissue inhibitor of metalloproteinases expression. Am J Respir Cell Mol Biol.

[12] Suganuma H, Sato A, Tamura R, Chida K (1995). Enhanced migration of fibroblasts derived from lungs with fibrotic lesions. Thorax.

[13] Li Y, Jiang D, Liang J, Meltzer EB, Gray A, Miura R (2011). Severe lung fibrosis requires an invasive fibroblast phenotype regulated by hyaluronan and CD44. J Exp Med.

[14] Cai GQ, Zheng A, Tang Q, White ES, Chou CF, Gladson CL (2010). Downregulation of FAK-related non-kinase mediates the migratory phenotype of human fibrotic lung fibroblasts. Exp Cell Res.

[15] Xia H, Khalil W, Kahm J, Jessurun J, Kleidon J, Henke CA (2010). Pathologic caveolin-1 regulation of PTEN in idiopathic pulmonary fibrosis. Am J Pathol.

[16] White ES, Thannickal VJ, Carskadon SL, Dickie EG, Livant DL, Markwart S (2003). Integrin alpha4beta1 regulates migration across basement membranes by lung fibroblasts: a role for phosphatase and tensin homologue deleted on chromosome 10. Am J Respir Crit Care Med.

[17] White ES, Atrasz RG, Hu B, Phan SH, Stambolic V, Mak TW (2006). Negative regulation of myofibroblast differentiation by PTEN (Phosphatase and Tensin Homolog Deleted on chromosome 10). Am J Respir Crit Care Med.

[18] Zhang J, Zhang P, Wei Y, Piao HL, Wang W, Maddika S (2013). Deubiquitylation and stabilization of PTEN by USP13. Nat Cell Biol.

[19] Vancheri C, Failla M, Crimi N, Raghu G (2010). Idiopathic pulmonary fibrosis: a disease with similarities and links to cancer biology. Eur Respir J.

[20] Yamada KM, Araki M (2001). Tumor suppressor PTEN: modulator of cell signaling, growth, migration and apoptosis. J Cell Sci.

[21] Jablonska E, Markart P, Zakrzewicz D, Preissner KT, Wygrecka M (2010). Transforming growth factor-beta1 induces expression of human coagulation factor XII via Smad3 and JNK signaling pathways in human lung fibroblasts. J Biol Chem.

[22] Xu X, Wan X, Geng J, Li F, Yang T, Dai H (2013). Rapamycin regulates connective tissue growth factor expression of lung epithelial cells via phosphoinositide 3-kinase. Exp Biol Med (Maywood).

[23] Xu X, Wan X, Geng J, Li F, Wang C, Dai H (2013). Kinase inhibitors fail to induce mesenchymal-epithelial transition in fibroblasts from fibrotic lung tissue. Int J Mol Med.

[24] Irizarry RA, Hobbs B, Collin F, Beazer-Barclay YD, Antonellis KJ, Scherf U (2003). Exploration, normalization, and summaries of high density oligonucleotide array probe level data. Biostatistics.

[25] Wright GW, Simon RM (2003). A random variance model for detection of differential gene expression in small microarray experiments. Bioinformatics.

[26] Ruthenborg RJ, Ban JJ, Wazir A, Takeda N, Kim JW (2014). Regulation of wound healing and fibrosis by hypoxia and hypoxia-inducible factor-1. Mol Cells.

[27] Lekkerkerker AN, Aarbiou J, van Es T, Janssen RA (2012). Cellular players in lung fibrosis. Curr Pharm Des.

[28] Cronkhite JT, Xing C, Raghu G, Chin KM, Torres F, Rosenblatt RL (2008). Telomere shortening in familial and sporadic pulmonary fibrosis. Am J Respir Crit Care Med.

[29] Chilosi M, Poletti V, Zamo A, Lestani M, Montagna L, Piccoli P (2003). Aberrant Wnt/beta-catenin pathway activation in idiopathic pulmonary fibrosis. Am J Pathol.

[30] Nho RS, Xia H, Diebold D, Kahm J, Kleidon J, White E (2006). PTEN regulates fibroblast elimination during collagen matrix contraction. J Biol Chem.

[31] Stambolic V, Suzuki A, de la Pompa JL, Brothers GM, Mirtsos C, Sasaki T (1998). Negative regulation of PKB/Akt-dependent cell survival by the tumor suppressor PTEN. Cell.

[32] Tamura M, Gu J, Matsumoto K, Aota S, Parsons R, Yamada KM (1998). Inhibition of cell migration, spreading, and focal adhesions by tumor suppressor PTEN. Science.

[33] Xia H, Diebold D, Nho R, Perlman D, Kleidon J, Kahm J (2008). Pathological integrin signaling enhances proliferation of primary lung fibroblasts from patients with idiopathic pulmonary fibrosis. J Exp Med.

[34] Liu Y, Soetandyo N, Lee JG, Liu L, Xu Y, Clemons WM (2014). USP13 antagonizes gp78 to maintain functionality of a chaperone in ER-associated degradation. Elife.

[35] Scortegagna M, Subtil T, Qi J, Kim H, Zhao W, Gu W (2011). USP13 enzyme regulates Siah2 ligase stability and activity via noncatalytic ubiquitin-binding domains. J Biol Chem.

[36] Araneda OF, Tuesta M (2012). Lung oxidative damage by hypoxia. Oxid Med Cell Longev.

[37] Mizuno S, Bogaard HJ, Voelkel NF, Umeda Y, Kadowaki M, Ameshima S (2009). Hypoxia regulates human lung fibroblast proliferation via p53-dependent and -independent pathways. Respir Res.

[38] Papakonstantinou E, Karakiulakis G, Tamm M, Perruchoud AP, Roth M (2000). Hypoxia modifies the effect of PDGF on glycosaminoglycan synthesis by primary human lung cells. Am J Physiol Lung Cell Mol Physiol.

