# Supplementary figures and images for: Down-regulation of USP13 mediates phenotype transformation of fibroblasts in idiopathic pulmonary fibrosis
Source: Respir Res. 2015 Oct 9;16:124. doi: 10.1186/s12931-015-0286-3 (PMC4600336; doi:10.1186/s12931-015-0286-3)

A

| Sample   | NC 1 | NC 2 | NC 3 | IPF1 | IPF2 | IPF3 | IPF4 | IPF5 |
|----------|------|------|------|------|------|------|------|------|
| A260/280 | 2.08 | 2.09 | 2.04 | 2.02 | 2.07 | 2.07 | 2.05 | 2.09 |

B

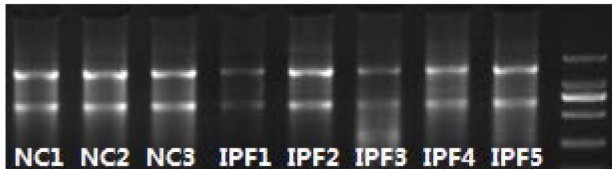

Supplement: Additional file 1: Figure S1. — The RNA purity and quality analysis. (A) The A260/A280 ratio of RNAs isolated from lung tissues of five IPF patients and three normal control subjects. (B) The representative image of the same RNAs showed by agarose gel electrophoresis. (PDF 903 kb) [file 12931_2015_286_MOESM1_ESM.pdf]
